# Supplementary material for: Structural basis for conductance through TRIC cation channels
Source: Nat Commun. 2017 May 19;8:15103. doi: 10.1038/ncomms15103 (PMC5477506; doi:10.1038/ncomms15103)
Supplement: Supplementary Information — Supplementary Figures, Supplementary Tables. [file ncomms15103-s1.pdf]

Supplementary Table 1 | Parameters for Calculation of Sequence Conservation

	Prokaryotic TRICs	Eukaryotic TRICs
Homolog search algorithm	PSI-BLAST	PSI-BLAST
Number of iterations	5	5
E-value cutoff	0.0001	0.0001
Proteins database	UNIREF-90	UNIREF-90
Maximum homologs to collect	500	500
Maximal %ID between sequences	95	95
Minimal %ID for homologs	35	35
Alignment method	MAFFT	MAFFT
Method of calculation	Bayesian	Bayesian
Model of substitution for protein	Best fit	Best fit
Query Sequence (gi Number)	499597352 (*Sa*TRIC)	74733603 (*Hs*TRIC-A)
Total unique sequences for calculation	100	169

Supplementary Table 2. Summary table for crystallization and structural analyses of *Sa*TRIC and *Cp*TRIC

Protein name	*Sa*TRIC				*Cp*TRIC
**Crystal name**	Type 1	Type 2a	Type 2b	Type 3	Se-Met
**Molecular Construct**	1-207,<br>L148M/L180M	1-207,<br>WT,	1-207,<br>WT,	1-207,<br>WT,	1-219,<br>WT,
**Protein type**	Se-Met	Native	Native	Native	Se-Met
**Protein Solution**	HEPES 20mM pH7.5,<br>NaCl 200mM,<br>DM 4mM	HEPES 20mM pH7.5,<br>NaCl 200mM,<br>DM 4mM	HEPES 20mM pH7.5,*<br>RbCl 200mM,<br>DM 4mM	HEPES 20mM pH7.5,<br>NaBr 200mM,<br>DM 4mM	HEPES 20mM pH7.5,<br>NaCl 200mM,<br>OM 40mM
**Wavelength**	0.97853 Å	0.97876 Å	0.81530 Å	0.91532 Å	0.97876 Å
**Space group**	P321	$P6_{(3)}$	$P6_{(3)}$	R32	R32
**Resolution**	~3.1 Å	~1.6 Å	~1.8 Å	~2.4 Å	~2.4 Å
**Solution for crystallization**	PEG200 40%,<br>NaCl 100mM,<br>$MgCl_2$ 100mM,<br>MES pH 6.0 100mM	PEG200 40%，<br>Li_2SO_4 100mM,<br>Na-Citrate pH 5.5 50 mM	PEG200 38%,<br>RbCl 100mM,<br>$MgCl_2$ 100mM,<br>HEPES pH 7.0 100mM,	PEG200 40%<br>Li_2SO_4, 100mM,<br>HEPES pH 7.0 100mM	PEG400 38%,<br>NaCl 100mM,<br>$CdCl_2$ 100mM,<br>Tris-HCl, pH8.5, 100mM,
**Method for crystallization**	LCP	LCP	LCP	LCP	Detergent micelle
**Beamline Facility**	SSRF BL19U1	SSRF BL19U1	APS 24IDE	SSRF BL19U1	SSRF BL19U1
**See a string of water molecules within the ion translocation pore?**		Yes, 9 water molecules	Yes, 9 water molecules	Unmodelled density	Yes, 6 water molecules
**Ion binding**		2 Na^{+}, along the trimeric three-fold axis	1 Mg^{2+}, along the trimeric three-fold axis	Not determined	4 Cd^{2+}
**Conformation for the N-THB (TM1-3)**		Unlocked	Unlocked	Unlocked	Unlocked
**Conformation for the C-THB (TM4-6)**		Locked	Locked	Unlocked	Locked
**Conformation of ion translocation pore**		Closed	Closed	Open	Closed

* The pH for the HEPES buffer was adjusted by using NH_4OH.

a.

```
                 TM1/TM4                 TM2/TM5            H23/H56     TM3/TM6
SaTIRC-N   7 IVFDIFNYIGIVAFAISGAIKAVKKGMDLLGVLVLGFSTALGGGIISNLLLGKTPPTNLIYYPYPITAFLASLATFVFYRIF
SaTIRC-C  93 KPLLYADAIGLGAFASSGASLAYSVSNNVILVVIVGAITAVGGGVIRDILSNE-VPLILTREFYATTAVIGSFVYFIASDL-
              :   : **: *** ***  *  .  . ::: *:::*  **:***:* ::* .: .*   *    *. **.:.*:. *:   :
                          1: SaTRIC-N     100.00    31.25
                          2: SaTRIC-C      31.25   100.00

                 TM1/TM4                 TM2/TM5            H23/H56     TM3/TM6
CpTIRC-N   1 MNDFLFYLDIFGVIVFALSGALMAGRYQLDPFGVVVLASVTAVGGGTIRDVILQT-PVFWVEKPYYLYVILATAILTIVLIRQPKRI
CpTIRC-C  83 PKRFLLIADALGLALFAVLGTQKALYLGAPIPVAVVLGTITGIAGGMIRDVLCNVIPMILREEIYALAAMLGGSLFIILHGLNWNDT
             : **:   * :*: :**: *:   *           .***.::*.:.** ****: :. *::   *: * *  .:*. ::: *:   : :

                          1: CpTRIC-N      100.00    27.91
                          2: CpTRIC-C       27.91   100.00
```

b

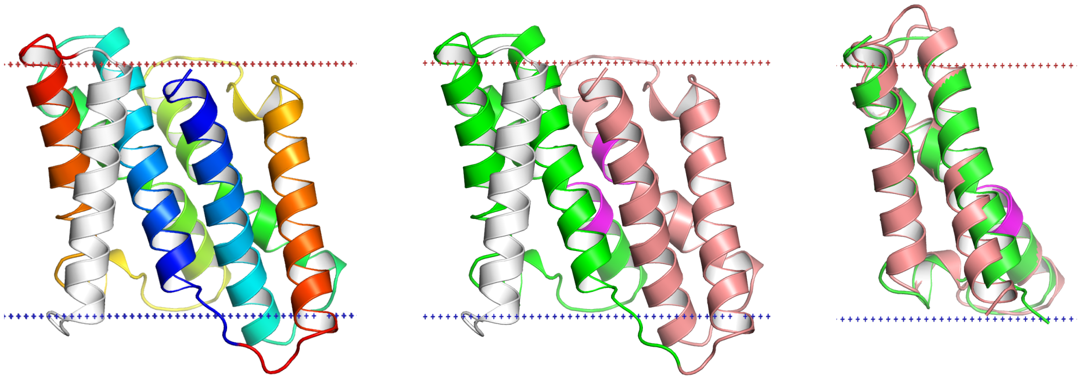

c

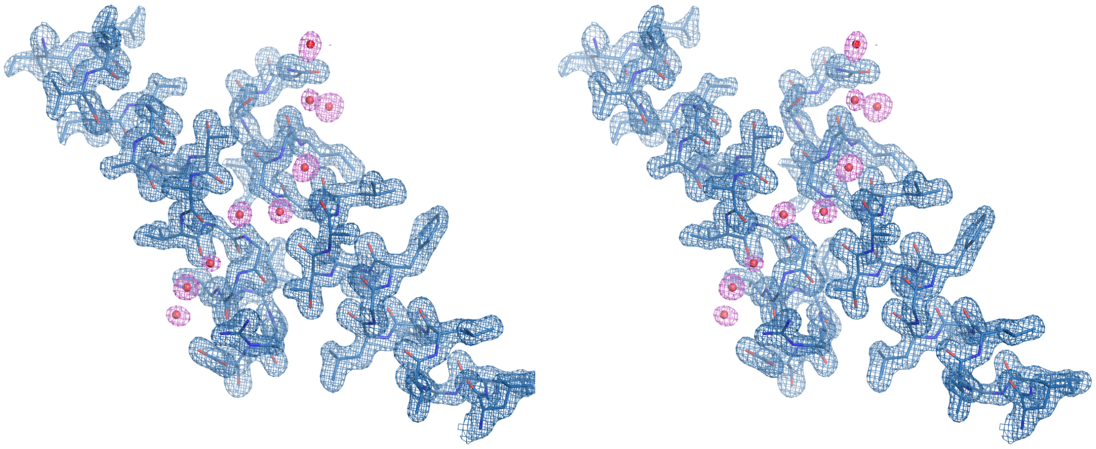

Supplementary Figure 1

The inverted repeats of triple-helix bundle (THB).

a. Sequence alignments of N-THB ($TM_{1\text{-}3}$) and C-THB ($TM_{4\text{-}6}$). Superior coils define extents of the *Sa*TRIC/CpTRIC helical segments; red letters mark residues that are involved in ion conduction pathway; **b.** Structural disposition of inverted repeat segments. Ribbon diagrams of a *Sa*TRIC protomer are viewed from within the membrane plane with different coloring schemes: (left) spectral coloring from dark blue at the N-terminus to red at the C-terminus for each THB and with grey for TM_7; (middle) N-THB in salmon and C-THB in green; (right) coloring as in the middle panel, but with N-THB superimposed onto the C-THB in the same orientation as in the middle. Membrane boundaries were calculated by OPM (Orientations of Proteins in Membranes) server; **c.** Stereo view of the kinked TM_2 and TM_5 helices. The experimental 2Fo-Fc map at 1.6 Å resolution is superimposed onto the *Sa*TRIC model. Water molecules in the ion-conducting pore are shown with purple densities. All density contours are at 1.5 σ.

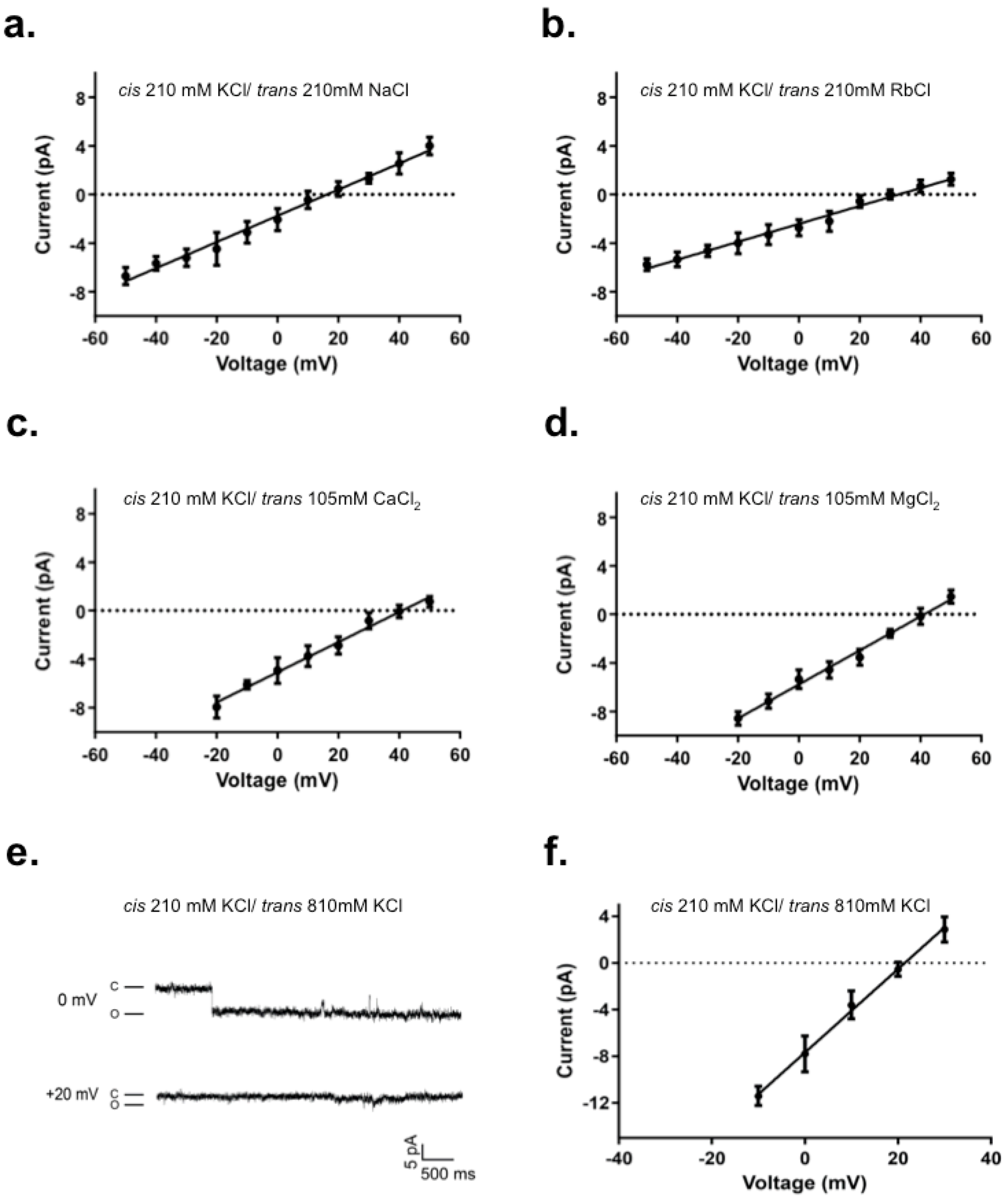

Supplementary Figure 2
The I-V plots and representative current traces for *Sa*TRIC wild type and D99A mutant

(**a-d)** The I-V plots for *Sa*TRIC wild type in different combination of solutions: **a**, I-V plot for *cis* 210 mM KCl/ *trans* 210mM NaCl; **b.** I-V plot for *cis* 210 mM KCl/ *trans* 210mM RbCl **c.** I-V plot for *cis* 210 mM KCl/ *trans* 105mM $CaCl_2$ **d.** I-V plot for *cis* 210 mM KCl/ *trans* 105mM $MgCl_2$. Data are presented as mean ± SEM (n=4 for each group). (**e)** Representative current traces of single *Sa*TRIC D99A mutant channel at 0 mV and +20 mV (210 mM KCl in *cis*- and 810 mM KCl in *trans*- chamber). The current of *Sa*TRIC D99A mutant channel is almost zero when the voltage is +20 mV. (**f**) Single *Sa*TRIC D99A mutant channel current-voltage relationship. Data are presented as mean ± SEM (n=3 for each point).

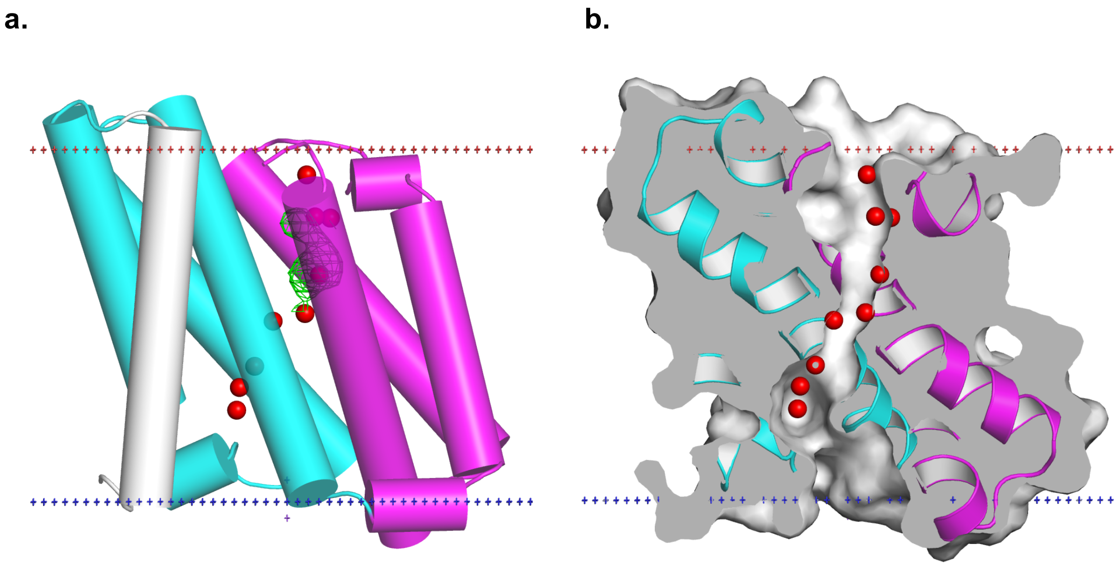

Supplementary Figure 3
Unmodelled density in the ion conducting pore (Type 3)

a, Cylindrical helices drawing of *Sa*TRIC structure (R32, Type 3), with N-THB in magenta, C-THB in cyan and TM_7 in white. A string of water molecules identified from the 1.8Å structure ($P6_3$, Type 2a) was superimposed into the structure (R32, Type 3), an unmodelled density was shown in green. **b,** Cross-section through the *Sa*TRIC(R32, Type 3). The model, cartoon presentation and water molecules are viewed as in a. Membrane boundaries were calculated as Fig. 2E.

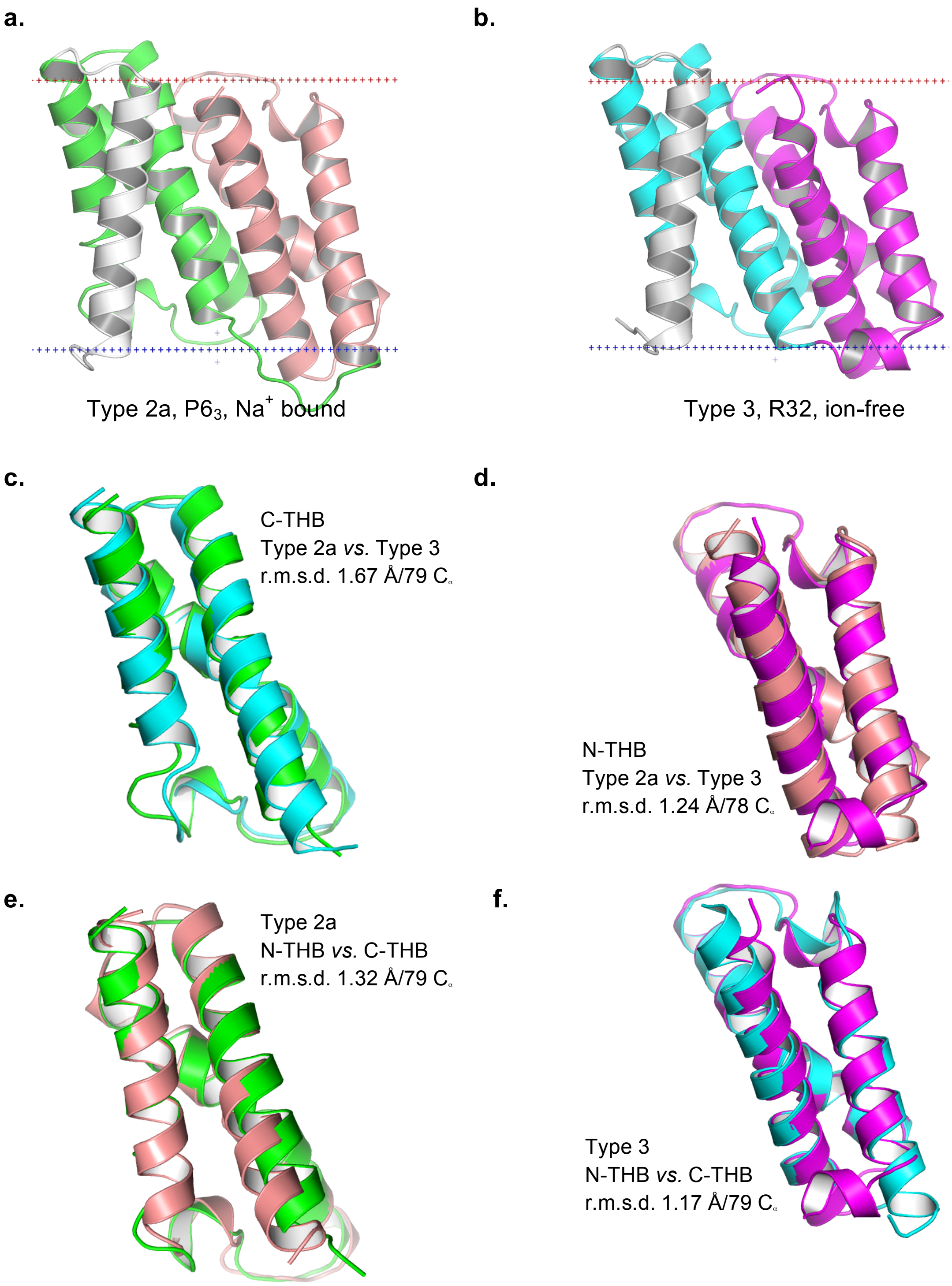

Supplementary Figure 4
Comparison of the structures from Type 2a and Type 3 crystals.

a, b, Ribbon diagram of the *Sa*TRIC structures: Type 2a, with N-THB in salmon, C-THB in green; Type3, with N-THB in magenta, C-THB in cyan; both TM7 in white. **c,** superimposed C-THB, Type 2a in green(P63) v.s. Type 3 in cyan (R32); **d,** superimposed N-THB, Type 2a in salmon(P63) v.s. Type 3 in magenta (R32); **e,** superimposed N-THB onto C-THB for Type 2a, N-THB in green and C-THB in salmon; **d,** superimposed C-THB onto N-THB for Type 3, N-THB in cyan and C-THB in magenta.

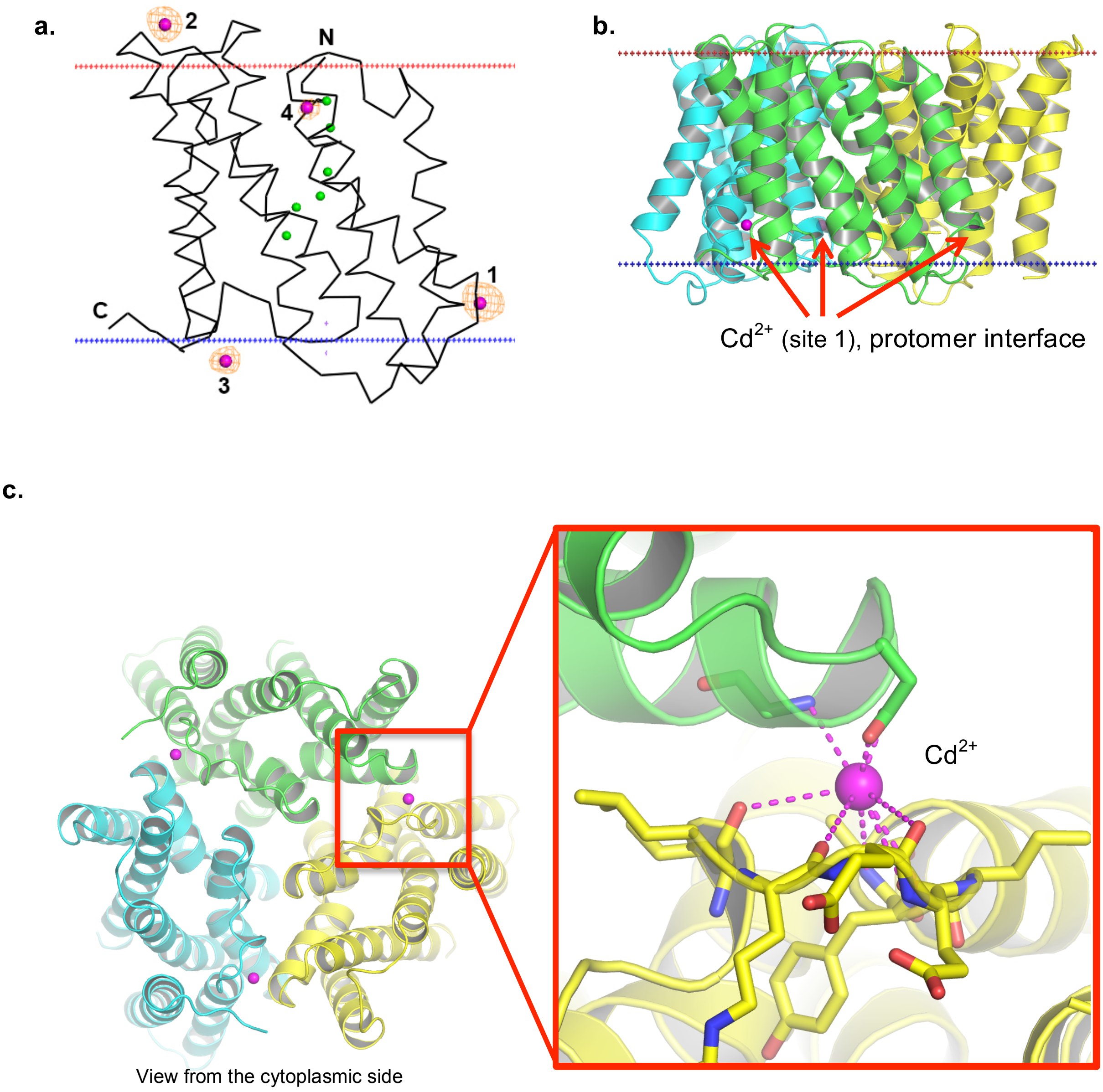

Supplementary Figure 5
Ion bindings in the prokaryotic *Cp*TRIC.

a, Cα backbone diagram of Cd^{2+} bound *Cp*TRIC protomer. Four bound Cd^{2+} ions are shown as purple spheres, Anomalous density contours are shown at 3.5 σ. Cd^{2+} at site 1 is located near the protomer interface, Cd^{2+} at site 2 is involved in molecular packing in the R32 lattice, Cd^{2+} at site 3 is near the cytoplasmic side and Cd^{2+} at site 4 is located at entrance of the ion conducting pore near the extracellular side. Water molecules within the ion conducting pore are shown as green spheres. **b,** Ribbons diagram of Cd^{2+} bound *Cp*TRIC trimer, with each protomer coloured in green, yellow and cyan. Cd^{2+} ion (site 1) bound to the protomer interface are shown as purple spheres. **c,** Left panel: Ribbon diagram of the *Cp*TRIC trimer, as in **a**, but viewed from cytoplasmic side. Right panel: A zoom-up view of the Cd^{2+} interacting network, residues involved in interaction with Cd^{2+} (site 1) are shown.

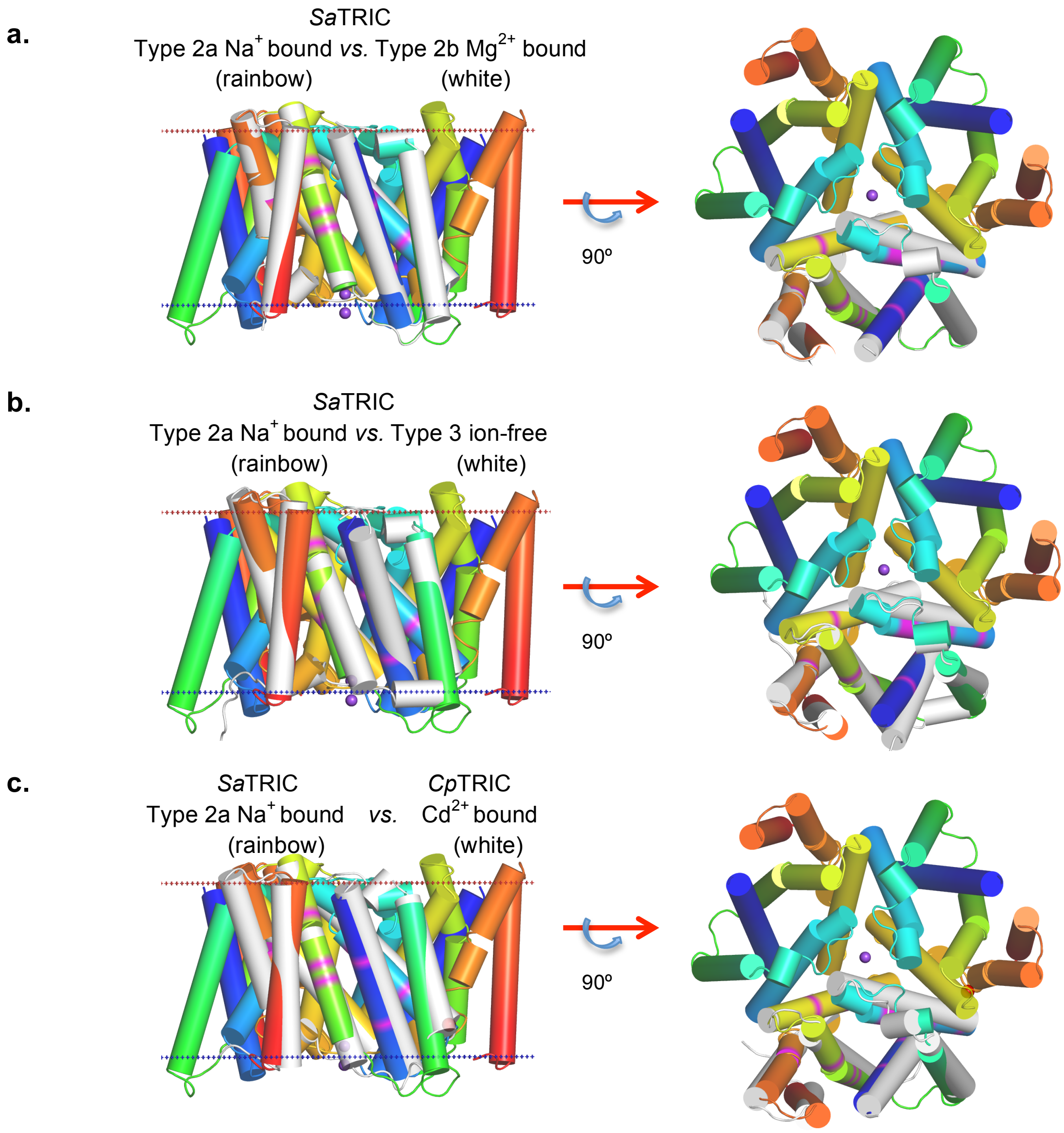

Supplementary Figure 6
Ion binding *v.s.* re-orientation of pore-forming helices.

a, Cylindrical helices drawing of Na^+ bound *Sa*TRIC trimer ($P6_3$, Type 2a), with each protomer coloured spectrally from dark blue at its N-terminus to red at its C-terminus. The Mg^{2+} bound structure (Type 2b) was superimposed, with only one protomer shown in grey. **b,** Cylindrical helices drawing of Na^+ bound *Sa*TRIC trimer ($P6_3$, Type 2a), with each protomer coloured spectrally from dark blue at its N-terminus to red at its C-terminus. The ion-free structure (Type 3) was superimposed, with only one protomer shown in grey. **a,** Cylindrical helices drawing of Na^+ bound *Sa*TRIC trimer ($P6_3$, Type 2a), with each protomer coloured spectrally from dark blue at its N-terminus to red at its C-terminus. The *Cp*TRIC structure (Se-Met) was superimposed, with only one protomer shown in grey. Cd^+ ions bound to Asp 40 near the protomer interface are shown as purple spheres.
Left panel: as view in the membrane; Right panel, as viewed from outside of the membrane. Membrane boundaries were calculated by OPM (Orientations of Proteins in Membranes) server, as in Fig. 2E.

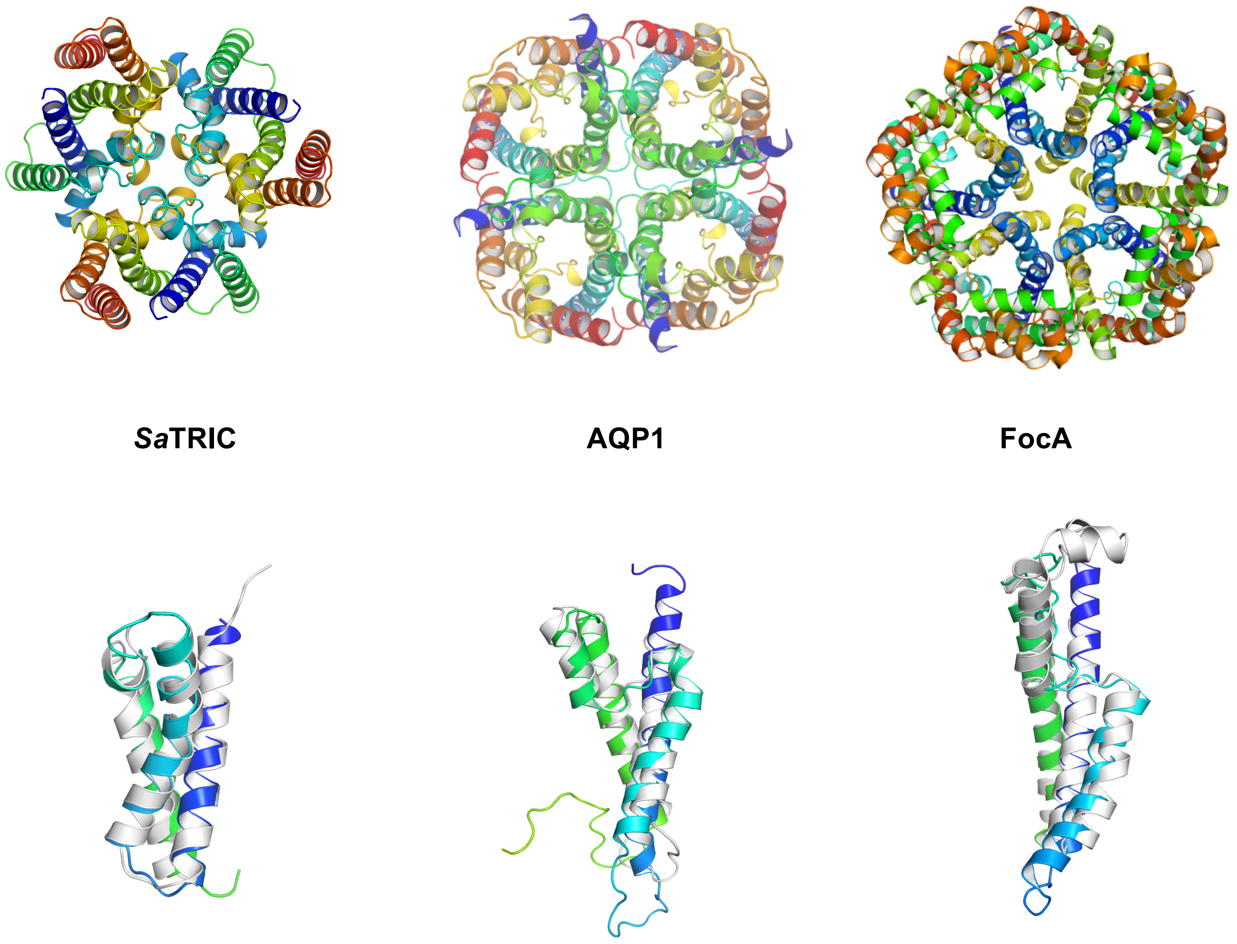

Supplementary Figure 7
Structural comparison of *Sa*TRIC, AQP1 and FocA

Upper panel：Ribbon diagram of *Sa*TRIC (trimer), AQP1 (tetramer) and FocA (pentamer), as viewed from the extracellular side, coloured spectrally from dark blue at its N-terminus to red at its C-terminus for each protomer.
Lower panel：Ribbon diagram of the superimposed structural comparison of $TM_{1\text{-}3}$, colored spectrally as in the upper panel, and $TM_{4\text{-}6}$, colored in white, for each protomer of *Sa*TRIC, AQP1and FocA.
